# Imapct of systems ambiguity on guideline compliance in intensive care units

**DOI:** 10.1186/1753-6561-5-S6-O48

**Published:** 2011-06-29

**Authors:** AP Gurses, Y Xiao, K Seidl

**Affiliations:** 1 Anesthesiology and Critical Care, Som, Johns Hopkins University, Baltimore, USA; 2 Baylor Health Systems, Dallas, TX, USA; 3 University of Maryland Medical Center, Baltimore, USA

## Introduction / objectives

Health care associated infections (HAI) in intensive care units (ICU) can be significantly reduced or eliminated by increasing care providers’ compliance with evidence-based guidelines. Using a human factors and systems engineering approach, we conducted a qualitative study to identify the underlying causes of non-compliance with evidence-based guidelines for preventing four types of HAI in ICUs.

## Methods

We conducted semi-structured, in-depth interviews with a total 20 surgical ICU care providers including three attending physicians, two residents, six nurses, three quality improvement coordinators, two infection control practitioners, two respiratory therapists and two pharmacists. Thematic analysis of the qualitative data was performed using a grounded theory approach.

## Results

A new framework called “systems ambiguity” that can be used to explain and prevent care providers' non-compliance with evidence-based guidelines emerged from the data. We define systems ambiguity as “uncertainty or vagueness that may prevent a work system from achieving its purpose.” Five major types of ambiguity that can affect care providers' compliance behaviors have been identified: task ambiguity, responsibility ambiguity, expectation ambiguity, method ambiguity, and exception ambiguity.

## Conclusion

Systems ambiguity framework can be used to (1) identify the underlying causes of care providers’ non-compliance with guidelines aimed at preventing HAI, and (2) guide efforts for developing effective interventions aimed at improving compliance rates. Future research should focus on designing multi-faceted interventions based on the systems ambiguity framework and evaluating the impact of these interventions.

## Disclosure of interest

A. Gurses Grant/Research support from AHRQ (K Award) and National Patient Safety Foundation, Y. Xiao: None declared, K. Seidl: None declared.

